# Effect of Smear Clear and Some Other Commonly Used Irrigants on dislodgement resistance of Mineral Trioxide Aggregate to Root Dentin

**DOI:** 10.4317/jced.53411

**Published:** 2017-05-01

**Authors:** Mona Sadegh, Hasti Sohrabi, Mohamadjavad Kharazifard, Farzaneh Afkhami

**Affiliations:** 1 DDS, MSc, Department of Endodontics, Tehran University of Medical Sciences, International Campus, Tehran, Iran; 2 DDS, Tehran University of Medical Sciences, International Campus, Tehran, Iran; 3 DDS, PhD, Department of Epidemiology, Tehran University of Medical Sciences, Tehran, Iran

## Abstract

**Background:**

This study aimed to assess the push-out bond strength of mineral trioxide aggregate (MTA) to root canal dentin after irrigation with Smear Clear in comparison with 2.5% sodium hypochlorite (NaOCl), 2% chlorhexidine (CHX) and saline as commonly used root canal irrigants.

**Material and Methods:**

The coronal and mid-root areas of maxillary anterior teeth were horizontally sectioned into one-millimeter thick slices. The root canal lumen of dentinal slices was dilated using a diamond bur with 1.3 mm diameter. After the application of MTA, the samples were incubated in 100% humidity for 10 minutes and were then randomly divided into four groups (n=20) and immersed in Smear Clear, 2.5% NaOCl, 2% CHX and saline for 30 minutes. No irrigant was used for the control group (n=20). A wet cotton pellet was placed on the samples and after 48 hours of incubation, push-out bond strength was measured using a universal testing machine. The samples were evaluated under a stereomicroscope to determine the mode of failure. One-way ANOVA was used to assess statistical differences among the groups.

**Results:**

The control group showed the highest bond strength with significant differences with other groups (*P*<0.05). Among the experimental groups, the saline group had no significant difference with CHX (P=0.09) but it had significant differences with Smear Clear and NaOCl groups (*P*<0.05). No significant difference in bond strength to MTA was noted after irrigation with Smear Clear, CHX and NaOCl (*P*>0.05). Other pairwise comparisons showed no significant difference (*P*>0.05).

**Conclusions:**

Irrigation with Smear Clear, CHX and NaOCl did not cause a significant change in bond strength of MTA to dentin.

** Key words:**Root Canal Irrigants, push-out, Mineral Trioxide Aggregate, dentin.

## Introduction

Mineral trioxide aggregate is an ideal calcium silicate-based restorative material composed of hydrophilic particles, hydrated calcium silicate gel and calcium hydroxide (1,2). It has optimal sealing ability, biocompatibility and insolubility, excellent adhesion to dentin and radiopacity (2). Moreover, it induces the regeneration of root cementum and dentin (3). It is also used for repair of root perforations during endodontic treatment (4). Depending on the time of occurrence of root perforation in terms of the endodontic treatment phase, it must be repaired prior to finalizing the endodontic treatment in order to improve prognosis (5,6). The most important shortcoming of MTA is its long setting time (4). Thus, its bond to dentin may be compromised by root canal irrigation with irrigating solutions (1).

Recently, a new formulation of ethylenediaminetetraacetic acid (EDTA) was introduced to the market with the brand name “Smear Clear” (SybronEndo, Orange, CA), which contains 17% EDTA, Cetrimide and a specific surfactant. It has higher wettability than the conventional EDTA and the manufacturer claims that it has higher cleaning efficacy than EDTA as well (7). Dunavant *et al.* (8) showed that Smear Clear had significant antibacterial activity against *Enterococcus faecalis* and had greater efficacy for elimination of biofilm than 2% CHX. Jantarat *et al.* (7) demonstrated that the opening of dentinal tubules was greater in the Smear Clear group; this indicated more efficient penetration of Smear Clear compared to other irrigants into dentinal tubules.

Çelik *et al.* (2) reported that irrigants did not compromise the push-out bond strength of calcium silicate cements to root dentin. Calcium silicate cements are currently used in endodontic procedures due to having optimal properties. However, Yan *et al.* (9) stated that the push-out bond strength of materials used for root perforation repair such as MTA depended on the solution in which they were immersed. Loxely *et al.* (10) reported that the bond strength of MTA significantly decreased following treatment with sodium perborate + saline or sodium perborate + superoxol. Bond strength of restorative materials to dentinal walls is an important factor in assessment of their sealing ability under functional masticatory forces and biomechanical loads as well as the treatment success (2). The push-out bond strength test is commonly performed for assessment of the push-out bond strength of root canal filling materials (4).

According to Collares *et al.*, (11) several factors can affect the results of push-out bond strength testing such as the storage time of samples, load velocity of the testing machine, use of human or bovine dentin and the part of tooth to be tested. Smear layer removal was the only factor that did not affect the push-out bond strength in the study by Collares *et al.* (11). Pane *et al.* (12) concluded that the push-out test might be suitable for ranking of root canal filling materials in terms of their efficacy.

This study sought to assess the effect of Smear Clear and some other commonly used irrigants on push-out bond strength of MTA to dentin *in vitro*.

## Material and Methods

Forty-eight extracted human single-rooted teeth were used in this study. The crowns were cut below the cementoenamel junction. The roots were mounted in a cylindrical metal mold measuring 35×25×10mm containing auto-polymerizing acrylic resin (ACROPARS, Marlic Medical industry, Iran) and the resin was allowed to set. After polymerization, the mounted roots were separated from the mold and stored in saline.

The coronal and mid-root dentin was cut into one-millimeter thick sections by a low-speed CNC (Nemofanavaranepars, Tehran, Iran) saw under water irrigation. Next, the lumen of dentinal discs was standardized using a round diamond bur with 1.3 mm diameter.

A mixture of MTA (Angelus, Londrina, PR, Brasil) was prepared according to the manufacturer’s instructions. It was gradually applied to the root canal lumen of dentinal slices by a carrier (D&P, Forgeman, Pakistan) and condensed by an endodontic plugger (D&P, Forgeman, Pakistan). Moist gel-foam (Clinisponge, yucelmedical, Turkey) was placed beneath the dentinal slices to simulate periapical tissue conditions. The excess material on the surface of slices was removed by a scalpel. All samples were evaluated under a stereomicroscope at ×40 magnification (Olympus, SZ51, Taiwan). Possible cracks, defects or gaps between the material and dentinal wall were fixed. The samples were placed inside plastic bags along with moist gauze and placed in an incubator at 37°C and 100% moisture for 10 minutes for primary setting. The samples were then randomly divided into four groups (n=20) and immersed in 2.5% NaOCl (Chloraxid, Cerkamed, Polska), 2% CHX (FGM, Dentscare LTDA, Brazil), saline (Shahid Ghazi Pharmaceutical Co., Tehran, Iran) or Smear Clear (SybronEndo, Orange, CA, USA) for 30 minutes. Next, the samples were rinsed with copious distilled water and incubated at 37°C and 100% moisture for 48 hours. In the control group, wet cotton pellets were placed on the MTA for 48 hours in order for the MTA to set (n=20).

**Figure 1:**
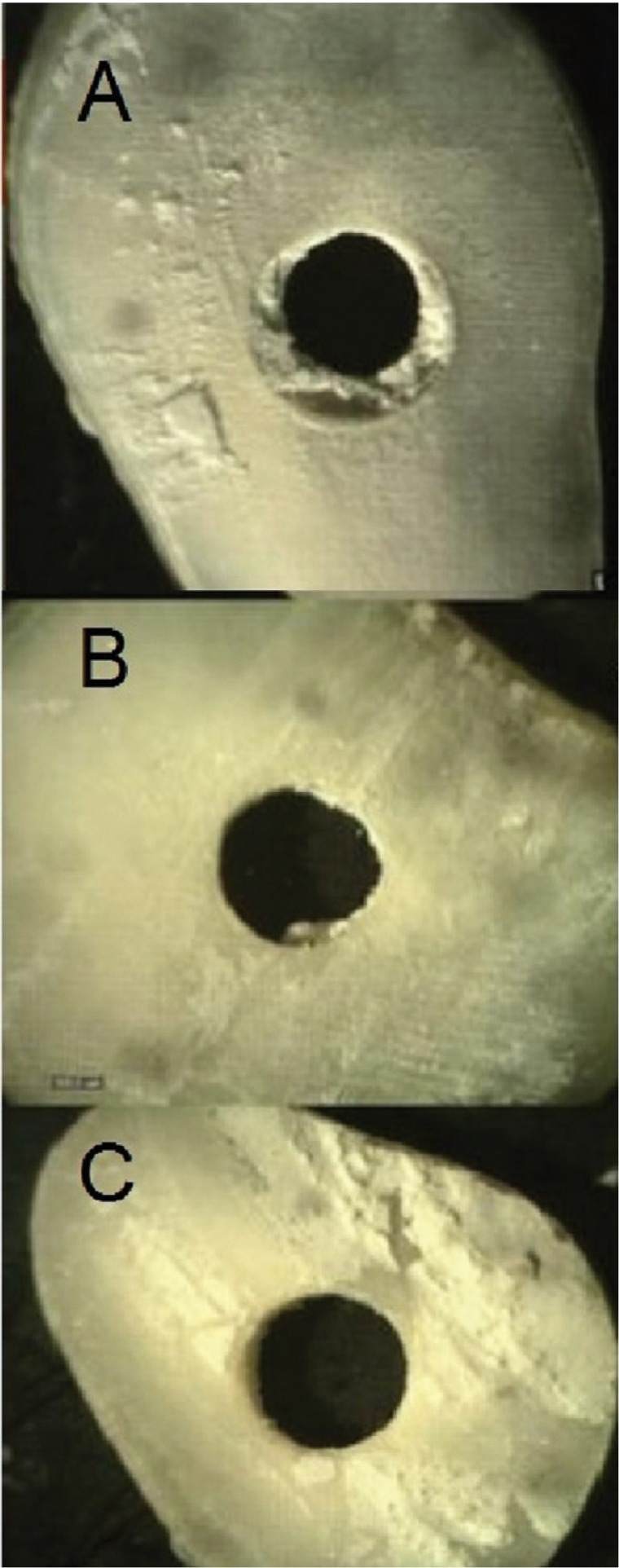
Microscopic micrographs of samples at ×10 magnification and mode of failure; A) Cohesive failure within MTA; B) Mixed failure, note the MTA residues in the root canal; C) Adhesive failure.

-The push-out bond strength testing:

The push-out bond strength was measured by a universal testing machine (STM-20, Santam machine, Germany). The samples were placed on a metal slab with a central hole to allow free movement of probe. Compressive load was applied vertically at a crosshead speed of 1mm/min. The probe had approximately 0.2 mm distance from the dentinal wall margin in order to be in contact with the material only. Maximum load at failure was recorded in Newton (N). The push-out bond strength was then calculated in Megapascals (MPa) using the formula N/2ᴫrh, where N is the maximum load at failure and ᴫrh is the bonding surface area. Mode of failure was determined under a stereomicroscope at ×40 magnification (Olympus, SZ51, Taiwan) and divided into three groups of adhesive (between the material and dentinal wall), cohesive within the material or dentin and mixed (a combination of adhesive and cohesive) (Fig. 1).

The data were analyzed using SPSS 22. One-way ANOVA and post-hoc test were used to compare the bond strength among the groups. Level of significance was set at *P*=0.05.

## Results

 Table 1 shows the mean and standard deviation of push-out bond strength in the groups. One-way ANOVA showed a significant difference in bond strength of the groups. The control group had the highest bond strength and showed statistically significant differences in this respect with Smear Clear (*P*=0.012), CHX (*P*=0.032) and NaOCl (P=0.006) groups. Also, significant differences were noted in bond strength between saline and Smear Clear (*P*=0.007) and saline and sodium hypochlorite (*P*=0.001) groups. Other pairwise comparisons did not show statistically significant differences (*P*>0.05).

**Table 1:**
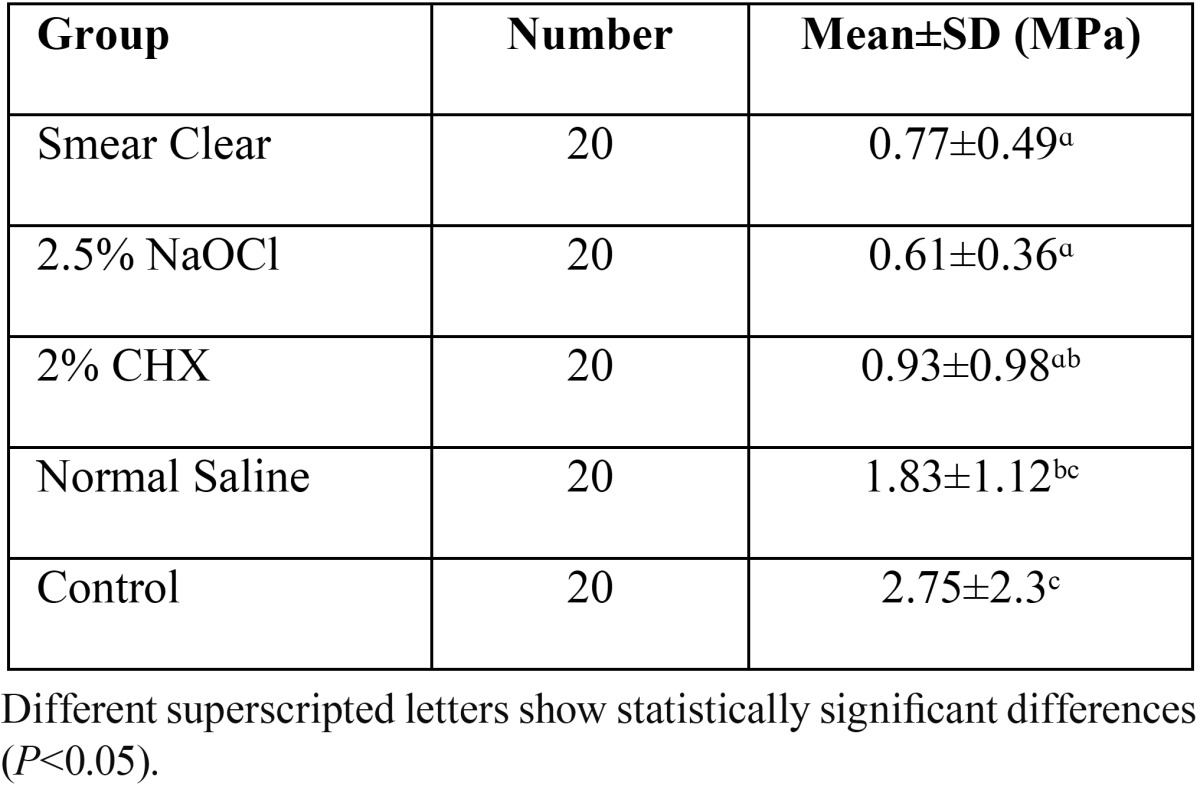
The mean push-out bond strength and standard deviation (SD) of the samples at 48 hours (MPa).

The mode of failure of samples are presented in  Table 2.

**Table 2:**
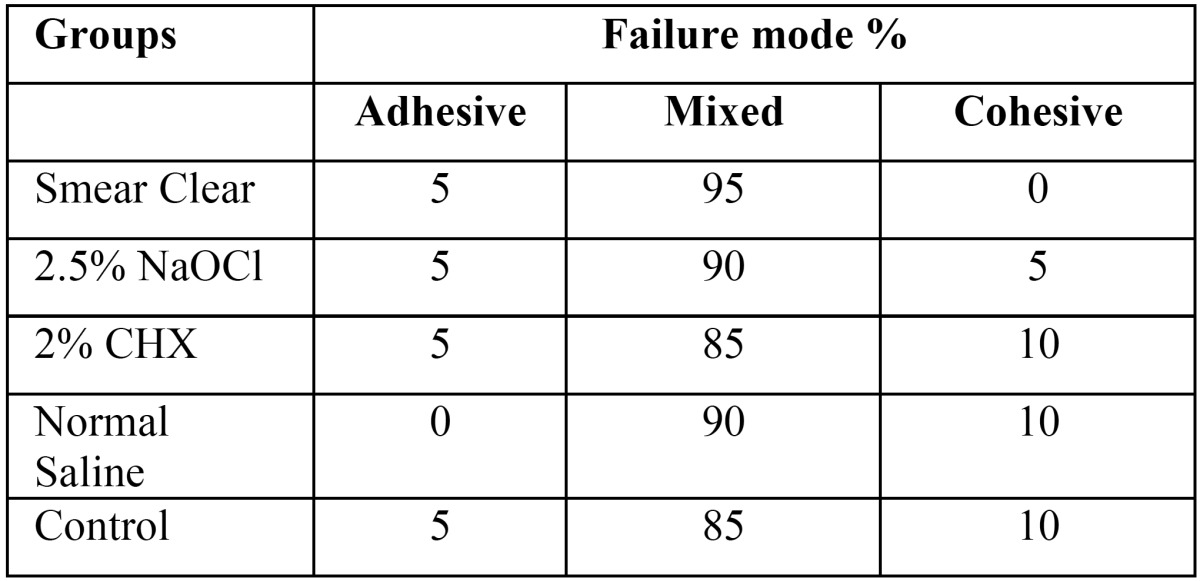
Mode of failure of the samples (%).

## Discussion

The gray MTA cement was first developed in Loma Linda University; however, its major drawback was causing tooth discoloration due to the presence of iron compounds in its formulation. By elimination of iron compounds, white MTA was developed (13) which was used in the present study.

Sodium hypochlorite solution can non-specifically dissolve collagenous and non-collagenous proteins and adversely affect the physical properties of dentin (14). It is the most commonly recognized antimicrobial irrigant in root canal therapy (15). Yan *et al.* (9) showed that bond strength of dentin to MTA decreased following the use of 5.25% NaOCl; however, the difference with the control group was not significant in this respect. In our study, the push-out bond strength of MTA to dentin was the lowest in 2.5% NaOCl group and it had a significant difference with the saline (control) group. This difference in the results of the two studies may be attributed to the different immersion periods of samples. In the study by Yan *et al.*, the samples were immersed for two hours in saline in the control group while we immersed the samples for 30 minutes in saline and incubated them for 48 hours. Thus, the time allowed for the setting of MTA in contact with saline in our study was longer than that in the study by Yan *et al.*, and this resulted in higher bond strength of MTA cement. In the study by Hong *et al.*, (1) non-accelerated MTA in contact with 2.5% NaOCl had lower push-out bond strength value than the control group, which was somehow in line with our findings. Nagas *et al.* (16) showed that MTA treated with 5.25% NaOCl had lower push-out bond strength than the control group. Another study (17) showed that samples treated with NaOCl had the lowest push-out bond strength value.

Chlorhexidine root canal irrigant has wide-spectrum antimicrobial activity against Gram-positive and Gram-negative bacteria. It has favorable substantivity and low cytotoxicity and thus, it is increasingly used in endodontics (18). Cecchin *et al.* (19) evaluated the effect of CHX and ethanol on the durability of the adhesion of fiber posts relined with composite resin to dentinal walls and showed that the group treated with CHX or ethanol showed equal bond strength along the root length after one day of immersion in water. They concluded that CHX and ethanol had no effect on the bond strength of fiber posts relined with composite resin to dentin. This finding was in contrast to our results, which may be due to the differences in the methodology of studies and the tested materials. Hong *et al.* (1) showed that CHX adversely affected the physical properties and hydration behavior of MTA. These findings were in agreement with our results since we showed that 2% CHX significantly decreased the push-out bond strength of MTA to dentin. Several studies demonstrated that bond strength of MTA significantly decreased when exposed to CHX (6,20,21).

Guneser *et al.* (21) indicated that MTA treated with saline had higher bond strength than the control MTA group; this finding was in contrast to our results since in our study, the push-out bond strength of the control group was higher than that of MTA treated with saline, but this difference did not reach statistical significance (*P*>0.05). Reyes-Carmona *et al.* (22) evaluated the biomineralization ability of MTA in presence of phosphate buffered saline (PBS) to increase its push-out bond strength to dentin and concluded that samples immersed in PBS had significantly higher bond strength at three days compared to those exposed to wet cotton pellets. Saline positively affects the size of MTA crystals (21) and completes the process of MTA hydration (23). Shokouhinejad *et al.* (24) evaluated the effect of acidic environment on push-out bond strength of MTA cement and concluded that MTA treated with PBS had significantly higher bond strength than the group treated with butyric acid; this shows that the pH of 7 is an ideal pH for the setting reaction of MTA to occur. Thus, placement of a cotton pellet dipped in saline on the MTA during its setting increases its bond strength. In the current study, saline-treated group ranked second after the control group in terms of the highest push-out bond strength.

Jantarat *et al.* (7) assessed the efficacy of the new formulation of EDTA (Smear Clear) for elimination of smear layer from the root canal dentin and showed that Smear Clear eliminated the smear layer from all coronal, middle and apical surfaces of the root but NaOCl was not capable of eliminating the smear layer from root canal surfaces. They concluded that Smear Clear had the highest efficacy for smear layer removal and cleaning of the root canal walls. Venghat *et al.*, in their *in vitro* study (25) revealed that Smear Clear was less efficient for smear layer removal than EDTA. Da Silva *et al.* (26) evaluated the efficacy of Smear Clear and EDTA for elimination of smear layer of permanent teeth after root canal instrumentation and concluded that Smear Clear had an efficacy similar to that of 14.3% EDTA for smear layer removal from the root canals, and the differences with the control group were statistically significant. To the best of our knowledge, no previous study has evaluated the effect of Smear Clear on push-out bond strength of MTA to dentin. In the current study, MTA treated with Smear Clear had significantly lower bond strength than the MTA treated with saline and the control group (*P*<0.05). Further studies are required to assess the effect of Smear Clear on hydration of MTA. Scanning electron microscopic analyses are also required to assess MTA cement after exposure to Smear Clear.

Mode of failure in all MTA groups in our study was evaluated under a stereomicroscope at ×40 magnification. Mode of failure was mixed in most samples ( Table 2), which was in contrast to the findings of Guneser *et al.*, (21) and Sobhnamayan *et al.* (6).This difference is due to the fact that in the study by Sobhnamayan *et al.*, (6) calcium enriched mixture cement was used, which is different from MTA in terms of the size of particles (22). In the study by Guneser et al., the powder/water ratio of MTA cement was 3:1, which was different from the powder/water ratio of MTA cement in the current study. In the study by Cecchin *et al.*, (19) mode of failure was mainly mixed in groups treated with CHX and saline (control), which was in line with our results.

## Conclusions

In the current study, the push-out bond strength of the MTA group exposed to saline was the highest. Immersion in Smear Clear, CHX and NaOCl irrigants decreased the bond strength of MTA to dentin following the first 10 minutes of its setting.
